# Prevalence and incidence of cancer amongst adults with intellectual disability — a systematic review and meta-analysis protocol

**DOI:** 10.12688/hrbopenres.13740.2

**Published:** 2024-02-02

**Authors:** Martin McMahon, Louise Lynch, Andrew Wormald, Jessica Eustace-Cook, Mary McCarron, Philip McCallion, Valerie Smith

**Affiliations:** 1 School of Nursing & Midwifery, The University of Dublin Trinity College, Dublin, D02 PN40, Ireland; 2 Trinity Centre for Ageing with Intellectual Disability (TCAID), The University of Dublin Trinity College, School of Nursing & Midwifery, Dublin, Ireland; 3 School of Social Work (College of Public Health), Temple University, Philadelphia, Pennsylvania, USA

**Keywords:** Intellectual disability, cancer, neoplasms, systematic review

## Abstract

**Background:**

People with intellectual disabilities have poorer health and die earlier than their peers without identified disabilities. This difference represents a significant inequality. Until recently, it was considered that cancer was less common in this population, mainly because they did not live long enough to develop age-related cancers. However, recent evidence has identified that people with intellectual disabilities may be at an increased risk of developing cancer but more likely to present for medical treatment at a later stage when cancer has spread. Nonetheless, the evidence is lacking and there is a need to understand the prevalence and incidence of cancer and subtypes of cancer in adults with intellectual disabilities.

**Methods:**

A systematic review and meta-analysis will be undertaken to investigate the prevalence and incidence of cancer and subtypes of cancer in adults with an intellectual disability. The JBI Systematic reviews of prevalence and incidence and the PRISMA-P (Preferred Reporting Items for Systematic Reviews and Meta-Analyses) guidelines were followed to develop this protocol. Electronic databases will be searched using predefined search terms to identify relevant studies using the Condition Context Population (CoCoPop) framework. Eligible studies should be observational and have published baseline data that have estimated or presented data on the prevalence or incidence of cancer in adults with intellectual disabilities. To assess the methodological quality of studies included in this review a modified version of the JBI Critical Appraisal Checklist for Studies Reporting Prevalence Data will be used. Prevalence and incidence proportions will be analysed separately with individual study data being pooled using the DerSimonian-Laird proportion method and a random effects meta-analysis will be undertaken.

**Discussion:**

This review will advance the epidemiological evidence to identify where targeted cancer care interventions are needed to help reduce the inequalities that this population experiences.

**Systematic review registration:**

PROSPERO registration number: CRD42023423584

## Introduction

Intellectual disability is a condition originating during the developmental period characterised by significantly below average intellectual functioning and adaptive behaviour ^1^. Internationally, the prevalence rate of intellectual disability is approximately 1–3% ^2^. People with intellectual disabilities are a vulnerable group with a complex health profile ^3^ that experience high levels of morbidity ^4^, mortality ^5, 6^ and general health inequalities ^7^ with regard to healthcare access and utilisation ^8, 9^, health surveillance ^10^, discriminatory attitudes ^11^ and diagnostic overshadowing ^12^. The consequence of such inequalities is well documented. For example, The Confidential Inquiry into Premature Deaths of People with Intellectual Disabilities in England revealed contributory causes to avoidable and untimely deaths in this demographic population ^13^. The inquiry found there is a substantial risk of premature mortality for people with intellectual disabilities, which may be attributed to untreated illnesses and deficiencies in the healthcare system for this population ^14^.

Inherent in the need to understand and address these inequalities that exist in this population, is the critical need to document and detail the diseases this population present with and die from. Historically, this has been challenging owing to diagnostic (for example often-atypical disease-related presentations) and methodological constraints (for example identifying people with intellectual disabilities). For this reason limited disease-related epidemiological evidence exists in this population. One noticeable disparity in the absence of literature concerns cancer. In Ireland, cancer is now the leading cause of death replacing heart disease ^15^. However, in the Irish intellectual disability population respiratory diseases are reported to be the leading cause of death ^16^. One plausible argument is that as cancer is a disease of old age ^17^ people with intellectual disabilities do not live long enough to be diagnosed with age-related cancers, notwithstanding the increases in their longevity over the last number of decades ^18^.

Nevertheless, there is some population level evidence emerging from Europe that identifies that people with intellectual disabilities are at an increased risk of developing any cancer, as well as several specific cancer types (e.g. cancers of the gastrointestinal system) ^19^ and are more likely to die from cancers than the general population ^20^. In contrast, a recent systematic review ^3^ examining the prevalence of physical health conditions in people with intellectual disabilities observed that solid cancers are likely to occur at the same or lower rates than the general population citing under-detection as being a likely factor. Under-detection of cancer in people with an intellectual disability is a particular concern. A recent English study linking data from the LeDeR mortality review ^21^ to cancer registries found that more than a third (35%; n=162) of decedents with intellectual disabilities and cancer, had cancer diagnosed via emergency presentations, with almost half (45%; n=228) of cancers at stage IV when diagnosed ^22^. In the same study, for colorectal cancers where pre-emptive screening was available, 43% died before reaching the colorectal screening age threshold. The findings of this study cited the absence of contemporary data about cancer in this population as being a particular issue.

Data about the prevalence and incidence of cancer diagnosis and mortality among people with intellectual disabilities are currently inconclusive. This review will determine how often people with an intellectual disability are diagnosed with cancer and what subtypes of cancer they are diagnosed with and die from. Although a relatively recent phenomenon in evidence synthesis, the prevalence and incidence systematic review and meta-analysis is an emerging methodology ^23^. Such reviews are becoming increasingly important as they can yield useful information on the burden of disease, illustrate trends, and inform geographical distributions of disease ^23, 24^. This information is useful and important to help shape the national and international landscape, particularly where there are gaps in the epidemiological data. From a national level, The Irish National Cancer Strategy (2017–2026) ^25^ has identified that the National Cancer Control Programme (NCCP) needs to focus on raising ‘cancer awareness and prevention initiatives’ and ‘prioritise disadvantaged populations and hard-to-reach groups’ (p.44). The cornerstone of the strategy outlines that early diagnosis will alter the landscape of cancer in Ireland by reducing mortality and improving survival. Nonetheless, such evidence is lacking in this population.

This proposed review and meta-analysis is part of a larger study that is being established called ‘The CANDID Study’. The CANDID study aims to explore **CAN**cer incidence, prevalence, **D**iagnosis, treatment, risk factors and outcomes in older adults with **I**ntellectual **D**isabilities in Ireland using longitudinal data from The Intellectual Disability Supplement to the Irish Longitudinal Study on Ageing (IDS-TILDA) ^26^, which was established as a supplement to the Irish Longitudinal Study on Ageing (TILDA) ^27^. The ability to use descriptive and longitudinal methods to examine cancer in older adults with intellectual disabilities will provide information critical to the provision of cancer care, and the development of national guidance for this population which is lacking nationally and internationally. The findings from this review will, in part support the NCCP in revealing inequalities that may exist and highlight where there is an urgent need to tailor cancer screening and prevention approaches for individuals with intellectual disability in Ireland.

### Aim of the protocol

To describe a protocol for a systematic review which will synthesise the available evidence on the prevalence and incidence of cancer in adults with intellectual disability.

The objectives of the review are to:

1. estimate the prevalence and incidence of any cancera in adults with intellectual disability.
2. estimate the prevalence and incidence of subtypes of cancers in adults with intellectual disability.
3. identify any trends in prevalence and incidence in subgroups of adults with intellectual disability based on i) gender (male versus female), ii) age (18–39 years and 40 years and older), and iii) severity of intellectual disability (mild, moderate, severe and profound)

The protocol was methodologically designed using JBI guidance for Systematic reviews of prevalence and incidence ^28^ and the PRISMA-P (Preferred Reporting Items for Systematic Reviews and Meta-Analyses Protocols) ^29^ guidelines. The protocol is registered at the International Prospective Register of Systematic Reviews (PROSPERO; registration number: CRD42023423584). Supplementary files are hosted on Open Science Framework (OSF) and are available here.

## Methods

### Eligibility criteria

***Types of studies.*** This review will include studies that are observational (retrospective and cross sectional), and/or longitudinal that have estimated or presented data on the prevalence or incidence of cancer in adults with intellectual disability. This review will consider cohort studies that yield estimates of incidence (rates and proportions) and cross-sectional studies that yield estimates of prevalence ^30, 31^. There will be no geographical, date or language restriction applied. Google translate will be used to screen abstracts of non-English language studies, and if included after screening, the full record will be translated by Google translate for full text review. Google translate has been found to be a viable and accurate tool for translating non-English reviews with over 91% accuracy agreement reported in a recent review ^32^.

The numerator and denominator of the prevalence and incidence estimation fraction must be included in the studies. For example, for incidence the numerator is the number of new cases of cancer during a specified time interval and the denominator is the population at the start of the time interval. For prevalence, the numerator is the number of cases of cancer at a specified point of time and the denominator is the population at that time. If this data is not published or available, requests will be made to the corresponding author(s). Data from case reports, conference abstracts and experimental or quasi-experimental studies such as randomised controlled trials and controlled clinical trials, will not be included in this review.

***Types of participants.*** Eligible studies will report on adults (≥ 18 years) of any gender, ethnicity or socioeconomic status who are identified as having intellectual disability ^1^. For the purposes of this review, intellectual disability will be accepted if the study reports that a participant has a) a standardised intelligence quotient test which was two Standard Deviations (SD) below the mean or lower, or b) they are receiving or eligible to receive services for people with intellectual disabilities due to adaptive or social functioning deficits in the jurisdiction the study was undertaken. Deficits should have occurred during the developmental period. All studies that report on specific chromosomal abnormalities (for example Down Syndrome) will be investigated for the presence of intellectual disability.

Given the challenge of identifying individuals with intellectual disability in studies of representative populations, frequently as a result of the absence of a generally accepted operational definition of intellectual disabilities ^33^, no a priori sampling size benchmark will be established and only studies which meet the inclusion criterion will be pooled for analysis. Studies that do not report the results for those with intellectual disability and those that focus on conditions where intellectual disability cannot be assumed, such as cerebral palsy, will be disregarded. Additionally, representation of specific subgroups of people with intellectual disabilities (e.g. people with intellectual disabilities receiving cancer treatment in hospital) will also be excluded.

### Outcome

The primary outcomes will be:

- Prevalence of any type of cancer
- Incidence of any type of cancer

Cancer types reported in the included studies will be diagnosed by an appropriately qualified medical professional with or without classification, recorded in cancer registers or self-reported.

The secondary outcomes of interest will be:

- Prevalence and Incidence of subtypes of cancer

Subtypes of cancer will include diagnosed cancers coded according to the International Classification of Disease (ICD) coding system at the time the study was undertaken in any of the following categories: C00-C97 Malignant neoplasms; D00-D09 In situ neoplasms; D10-D36 Benign neoplasms and D37-D48 Neoplasms of uncertain or unknown behaviour (An example of these cancers is provided under *Extended data*). For meta-analyses based on cancer sub-type, two or more studies will need to report on the same cancer type.

### Search criteria, study selection and data extraction

A Subject Librarian (JEC) was engaged at the developmental stage of this review and with the primary author (MMcM) the Condition Context Population (CoCoPop) ^23^ framework was used to develop the search strategy ( Table 1). This framework is commonly used for review questions focusing on prevalence. The search strategy was initially built using EMBASE (Elsevier) and then adapted for the other databases, as listed below. Database thesauri were reviewed for controlled language and synonyms. A keyword list was developed and adapted with additional input from the primary author. The search will utilise a combination of database specific control language and keywords (these will remain the same for each database), which will be combined with the OR Boolean operand. To increase the sensitivity of the search, NEAR proximity operators will be applied to the keyword search. The proximity will require cancer keywords appearing within 6 words of patient keywords with a proximity of 4 from prevalence. Each concept search will be run independently, and then combined with the AND operand to provide the final set of records for screening. The search strategy was peer-reviewed by two additional Subject Librarians ^1^ and the Peer Review of Electronic Search Strategies (PReSS) checklist to improve quality of the literature search was used ^34^.

**Table 1.** CoCoPop for the proposed research questions.

**Condition**	Cancer
**Context**	Prevalence or incidence
**Population**	Adults with intellectual disability

The search is comprised of two key concepts:

Prevalence/Incidence of Cancer AND Intellectual Disability.

***Electronic database searching.*** Systematic searches will be undertaken in Embase (Elsevier; 1947-), MEDLINE (EBSCO; 1879-), CINAHL Ultimate (EBSCO; 1937-), PsycINFO (EBSCO; 1967-) and Web of Science – Core Collection (Clarivate; 1945-). No limits or filters will be placed on the search. Each database will be searched from inception to the search date. The reference lists from included full-text articles will be hand searched and forward citation searching will be undertaken.

The EMBASE search strategy is provided in Table 2. 

**Table 2.** Search string for electronic database Embase.

**Context & ** **Condition**	#1	‘cancer patient’/exp OR ('neoplasm'/exp AND 'patient'/exp) AND 'prevalence'/de
	#2	(oncolog* OR cancer* OR neoplasm* OR malign* OR carcin*) NEAR/6 (patient* OR client* OR individual* OR person* OR people*) NEAR/4 (prevalen* OR occur* OR incidenc*)
	#3	#1 OR #2
**Population**	#4	'mentally disabled person'/exp OR 'mental deficiency'/exp OR 'intellectual impairment'/exp OR 'cognitive defect'/exp
	#5	‘Intellectual Disabil*’:ab,ti OR ‘Intellectually disabled’:ab,ti OR ‘mentally disabled’:ab,ti OR ‘mental disabil*’ :ab,ti OR ‘Intellectual Development Disorder*’:ab,ti OR ‘mental handicap*’:ab,ti OR ‘mentally handicapped’: ab,ti OR ‘mentally impaired’:ab,ti OR ‘mental impairment*’:ab,ti OR ‘intellectual impairment*’:ab,ti OR ‘developmental disabil*’:ab,ti OR ‘mental defici*’:ab,ti OR ‘intellectual retard*’:ab,ti OR ‘mental retard*’: ab,ti OR ‘mentally retarded’:ab,ti OR ‘intellectually challenged’:ab,ti OR ‘intellectually deficient*’:ab,ti OR ‘intellectually handicapped’:ab,ti OR ‘intellectually impaired’:ab,ti OR ‘intellectually retarded’:ab,ti OR ‘mentally challenged’:ab,ti OR ‘mentally defici*’:ab,ti OR ‘cognitive disab*’:ab,ti
	#6	#4 OR #5
**Final**	#7	#3 AND #6

***Grey and other literature.*** In addition to the core database search, supplementary databases searches will be run. These will include but are not limited to Global Index Medicus (WHO), Cochrane Library, British Nursing Index and Proquest Dissertations and Theses. Grey Literature searching will be undertaken in national and institutional repositories, search engines, including Google Scholar and relevant health and intellectual disability websites. All relevant articles will be added to Covidence for screening. All results will be reported into a spread sheet and added as a supplementary file to the final publication for transparency.

***Software.*** Covidence will be used for screening. EndNote X20 by PDF Tron™ Systems Inc will be used to as a bibliography manager. Review Manager 5.4 and MetaXL software will be used for meta-analysis.

***Study screening and selection.*** All results from each database will be exported into EndNote 20 and an initial deduplication will occur. The resultant articles will then be exported for title and abstract screening into Covidence. To ensure that the eligibility criteria will be applied consistently by each of the screeners (MMcM, LL and AW) a pilot test on a sample of 20 records will be undertaken initially, with screening congruency and understanding assessed via discussion and consensus.

Three reviewers (MMcM, LL and AW) will independently screen all retrieved records against the review’s inclusion criteria on titles and abstracts initially and then on full text. Disagreements, if any arise, will be discussed between. If no consensus can be reached, the matter will be referred to a third reviewer (MMcC), who will make the final decision. This procedure will be carried out during each phase of the screening process. The screening and selection process for the study will be reported in accordance with the (PRISMA) 2020 updated guideline for reporting systematic reviews ^35^, and a PRISMA flow diagram will be generated.

***Study quality and risk of bias assessment.*** A modified version of the JBI Critical Appraisal Checklist for Studies Reporting Prevalence Data will be used to evaluate the included studies' methodological quality and establish the degree to which bias was addressed in the study's design, conduct, and analysis ^23^. This is a nine-question tool from the Joanna Briggs Institute that addresses the following:

- appropriateness of the sampling frame and study participants
- the adequacy of the sample size
- the description of study subjects and setting
- sufficiency of data analysis
- validity of identification of condition
- reliable measurement of condition
- appropriate statistical analysis
- response rate.

For each question there is a ‘yes’, ‘no’, ‘unclear’ or ‘not applicable’ response. Three reviewers (MMcM, LL & AW) will independently determine the quality of each included study where ‘yes’ and ‘not applicable’ are scored ‘2’, unclear scored ‘1’‘ and ‘no’ scored ‘0’. This will leave a range of scores of ‘0–18’ with 18 being the highest quality. If MMcM or AW have any disagreements in determining quality, this will be resolved through discussion. However, if consensus cannot be achieved a third reviewer (MMcC) will make the final decision. The final quality assessment score for each included study will be reported along with the overall median and IQR quality assessment scores.

***Data extraction.*** Data from the included studies (black and grey literature) will be extracted using a pre-designed data extraction form. A supplementary data extraction file in Microsoft Excel is available in OSF under *Extended data*. The extraction form will be piloted on five studies to make any refinements and finalised by MMcM, LL and AW. Data will be extracted by MMcM, LL and AW will undertake checks on half of the extracted studies. If there are major discrepancies identified, all the extracted records will be reviewed. Table 3 summarises the extracted items.

Where data is not presented or missing in the study the research team will contact the corresponding author(s) to request this. If the corresponding author(s) do not respond within 3 weeks, set with a reminder sent after one week , their study will be excluded from the review.

**Table 3.** Items extracted from included studies from literature search.

Authors and year
Grey or black literature
Country of study
Study aim
Sample characteristics
Sample origin
Age range (mean (SD); median)
Gender (%)
Degree of intellectual disability mild, moderate, severe or profound (%)
Method of cancer diagnosis
Subtype of cancer(s) as per ICD-Classification system
Death (%)
Cancer cases (n)
Sample size N
Cancer prevalence % (95% CI)
Cancer prevalence % (95% CI).

### Data analysis and synthesis

It is important to highlight that there is no gold standard for how data analysis in prevalence and incidence systematic reviews should be performed and reported. However, where achievable, meta-analyses will be conducted using Review Manager 5.4 for pairwise comparisons, and MetaXL will be used to pool overall incidence and prevalence. Following JBI guidance, if meta-analysis is not possible, narrative synthesis will be conducted as the primary mechanism of data synthesis with tables, graphs and figures presenting the results of the prevalence and the incidence of cancer and subtypes of cancer ^36^. Individual study data will be pooled using the DerSimonian-Laird proportion method ^37^ and a random effects meta-analysis will be undertaken ^36, 38^. Following guidance from the JBI and Cochrane Handbook for Systematic Reviews, meta-analysis of incidence and prevalence data of cancer and cancer subtypes will be undertaken separately. The Standard Error (SE) of the incidence and/or prevalence can be determined using the numerator and denominator of the prevalence and incidence estimation fractions. Using Forest plots, separated and pooled incidence and prevalence along with 95% confidence intervals will be plotted. Values of *p* < .05 will be considered statistically significant and results will be reported in accordance with PRISMA guidelines ^35^. The Q-statistic test (to inform about the presence of heterogeneity) and I2 statistic (to quantify the degree of heterogeneity), and their related 95% CIs, will be calculated to determine statistical heterogeneity of the incidence and prevalence values in the included studies ^39^. Following Guidance from Higgins *et al.* (2003) ^40^, the threshold for interpreting the degree of heterogeneity will follow these parameters:

▪ 0% to 40%: might not be important
▪ 30% to 60%: may represent moderate heterogeneity
▪ 50% to 90%: may represent substantial heterogeneity
▪ 75% to 100%: considerable heterogeneity

Where there is 60% or more heterogeneity, these studies will not be included in meta-analysis. Additionally, where there are at least ten studies included in the meta-analysis, funnel plots will be used to make a visual assessment of whether small-study effects are present.

Sensitivity analysis will also be undertaken. All sensitivity analysis will be reported in summary tables and made available as a supplementary file to accompany the completed systematic review.

Potential influence on prevalence and incidence estimates will be explored using sensitivity and subgroup analyses as follows:

Sensitivity analyses will be conducted based on

- study quality/risk of bias, with studies of QA scores outside of the upper quartile excluded from the analysis
- study design, with estimates of cancer prevalence from retrospective and prospective studies analysed separately to assess variation based on study design
- geographical region, based on study location and low-income countries, middle income countries and high-income countries

The variation in the prevalence and incidence estimates, where sufficient data are reported in the included studies, will be explored based on subgroup analyses of:

- gender: male versus females
- age: 18–39 years and 40 years and older
- severity of intellectual disability (mild versus moderate, mild versus severe, mild vs profound, moderate versus severe, moderate versus profound and severe versus profound)

## Study status

JEC and MMcM are currently in the process of undertaking the literature search.

## Patient and public involvement

Engagement with knowledge users and patient and public involvement (PPI) involving people with intellectual disabilities themselves has been central to the development of the ‘The CANDID Study’. The research team consulted with Trinity College Dublin PPI Ignite Office and spoke with people with intellectual disabilities and their family members. This has shaped the development of this systematic review protocol through identifying and prioritising the research question. Patient and public involvement will be an integral part of the study.

## Discussion

This planned review and meta-analysis will explore prevalence and incidence of cancer in adults with intellectual disability. As there is limited contemporary data about cancer in this population, this review will provide valuable information to help understand how common cancer is in adults with an intellectual disability and what type of cancers adults with an intellectual disability are being diagnosed with. Such information on cancer can be used to help target cancer inequalities that are believed to exist in this population and to improve outcomes.

## Dissemination of findings

The findings from this review will be submitted for publication in an Open Access cancer related health journal and be presented at national and international conferences.

The findings of this review will be used to inform the NCCP about the prevalence and incidence of cancers and trends of cancer in adults with intellectual disability. It will be used to help reach ‘marginalised and hard to reach groups’ aligned with the Irish Cancer Strategy ^25^.

## Conclusion

Following the JBI Systematic reviews of prevalence and incidence ^28^ and the PRISMA-P (Preferred Reporting Items for Systematic Reviews and Meta-Analyses) ^29^, this systematic review protocol describes the review methodology, eligibility criteria, methods of determining data quality, method for screening research papers for inclusion, methods of data extraction and the process for conducting data synthesis and meta-analysis to investigate the prevalence and incidence of cancer in adults with intellectual disability. It is believed this review will help synthesize the available evidence to identify cancer burden at specific points in time and over different points of time. It is anticipated that this review will help address the inequities that exist in the understanding of cancer and that the provision of cancer services that need to be delivered to this population

## Data Availability

OSF: Prevalence and incidence of cancer amongst adults with intellectual disability: a systematic review and meta-analysis. DOI 10.17605/OSF.IO/RUAQ5 This project contains the following extended data: Data Extraction Form Prevalence and incidence of cancer amongst adults with intellectual disability a systematic review and meta-analysis protocol.xlsx ICD Cancer Coding .xlsx Data are available under the terms of the Creative Commons Attribution 4.0 International license (CC-BY 4.0). OSF: Prevalence and incidence of cancer amongst adults with intellectual disability: a systematic review and meta-analysis. DOI 10.17605/OSF.IO/RUAQ5
