# Supplementary material for: Prevalence and incidence of cancer amongst adults with intellectual disability — a systematic review and meta-analysis protocol
Source: HRB Open Res. 2024 Feb 2;6:51. Originally published 2023 Sep 13. [Version 2] doi: 10.12688/hrbopenres.13740.2 (PMC10850849; doi:10.12688/hrbopenres.13740.2)
Supplement: Peer review history

# Reviewer report: Reviewer response for version 2

**DOI:** 10.21956/hrbopenres.15198.r37989

**Published:** 2024-02-07

**Authors:** Trine Allerslev Horsbøl

**Affiliations:** 1 National Institute of Public Health, University of Southern Denmark, Copenhagen, Denmark

Congratulations to the authors on their great job. I have no further comments, and I am looking forward to reading the results of the systematic review.

Is the study design appropriate for the research question?

Yes

Is the rationale for, and objectives of, the study clearly described?

Yes

Are sufficient details of the methods provided to allow replication by others?

Yes

Are the datasets clearly presented in a useable and accessible format?

Not applicable

Reviewer Expertise:

Cancer, intellectual disability, diabetes, epidemiology, systematic reviews, public health

I confirm that I have read this submission and believe that I have an appropriate level of expertise to confirm that it is of an acceptable scientific standard.

# Reviewer report: Reviewer response for version 1

**DOI:** 10.21956/hrbopenres.15030.r36980

**Published:** 2023-11-27

**Authors:** Owen Doody

**Affiliations:** 1 Department of Nursing and Midwifery, North Bank Campus, University of Limerick (UL), Limerick, Ireland

This is a clear and relevant protocol with minor comments and rational needed.

 General point using people with intellectual disability and people with intellectual disabilities - can one term be used consistently throughout the paper.

 Introduction - cancer under detected and die before reaching screening age mentioned -- I am wondering is there evidence of a post mortem being performed and how this might relate to existing figures? Introduction ends with the proposed review and links to IDS-TILDA and could be relooked at in terms of how related to evidence presented in the introduction and the gap that exists and will be filled by this review and acknowledge part of a wider study. 

    

 Aim - age group 18-39 and 40+ given the age profile of ID should consideration be given to subdividing the 40+ age group.   

 Methods - why not under 18 given Down’s Syndrome and leukemia justify adults only versus a life approach? Why use a modified version of the JBI critical appraisal tool and what modification are you using or is it a previous modification you are choosing? Also - intellectual disability will be accepted if the study reports that a participant has a standardised intelligence quotient test which was two Standard Deviations (SD) below the mean or lower, or they are receiving or eligible to receive services for people with intellectual disability due to adaptive or social functioning deficits in the jurisdiction the study was undertaken and deficits should have occurred during the developmental period - how will you address / intelligence quotient test or definition of ID in papers or will you accept paper that identify population as having an ID even if a definition or IQ is not reported also are you assuming deficits should have occurred during the developmental period if person is receiving a service -  greater clarity here is required.

 The protocol is overall described well and the planned work is important and relevant.

Is the study design appropriate for the research question?

Yes

Is the rationale for, and objectives of, the study clearly described?

Yes

Are sufficient details of the methods provided to allow replication by others?

Yes

Are the datasets clearly presented in a useable and accessible format?

Yes

Reviewer Expertise:

Intellectual disability

I confirm that I have read this submission and believe that I have an appropriate level of expertise to confirm that it is of an acceptable scientific standard.

# Reviewer report: Reviewer response for version 1

**DOI:** 10.21956/hrbopenres.15030.r36134

**Published:** 2023-10-10

**Authors:** Trine Allerslev Horsbøl

**Affiliations:** 1 National Institute of Public Health, University of Southern Denmark, Copenhagen, Denmark

Thanks to the authors for submitting this protocol. In a detailed way, they describe the rationale and methodology of a review on prevalence and incidence of cancer among adults with intellectual disability.  

 I think the planned work is extremely important and relevant, and I am looking forward to seeing and using the results.

 I have some minor comment to the protocol.

1. Overall I suggest that the authors make a thorough linguistic revision of the paper. As it is now, there are a lot of long sentences, and a lot of commas are missing. Further, there are several words that should be combined with a hyphen. For example age-related, disease-related, under-detection etc. Also, in some sentences, words are missing, e.g. *Two reviewers (MMcM and AW) will independently screen all retrieved records against the review’s inclusion criteria.* Or wrong: *Cancer prevalence % (95% CI) and Cancer prevalence % (95% CI) *and *Where data is not presented or missing in the research study *(delete research).
2. It is stated that eligible studies have published baseline data that have estimated baseline data on incidence and prevalence of cancer. Incidence can not be estimated from baseline data, as it includes follow-up. I therefore suggest that you reconsider this inclusion criteria.
3. Inclusion criteria of study designs needs to be sharpened. A retrospective design is most often a case-control study, and this design is not relevant when estimating prevalence or incidence. Prevalence is most often estimated in a cross-sectional study, and incidence is measured in a cohort study. Thus, in my point of view, these two designs are the relevant designs to consider.
4. Please specify, if you only consider studies that report incidence proportions (that is the frequency measure that is described as it is now, with number of persons in the denomoniator). Do you exclude studies that report incidence rates? (new cases pr. number of person-years).
5. You only consider studies, where intellectual desibility is defined through a standardised intelligence quotient or receiving services for people with intellectual disability. What about diagnoses? For example on intellectual disability, Down Syndrome etc.? This is often the way that people with intellectual disabilities are identified in register-based studies.
6. I think you should clarify the same for cancer diagnoses. Is it okay with cancer cases registered in Cancer Registers?
7. You state that *if no consensus can be reached, the matter will be referred to MMcC, who will make the final decision. *Have you considered including a third person in these discussions instead?

Is the study design appropriate for the research question?

Yes

Is the rationale for, and objectives of, the study clearly described?

Yes

Are sufficient details of the methods provided to allow replication by others?

Yes

Are the datasets clearly presented in a useable and accessible format?

Not applicable

Reviewer Expertise:

Cancer, intellectual disability, diabetes, epidemiology, systematic reviews, public health

I confirm that I have read this submission and believe that I have an appropriate level of expertise to confirm that it is of an acceptable scientific standard, however I have significant reservations, as outlined above.
